# SIRT1 Regulates Endothelial Notch Signaling in Lung Cancer

**DOI:** 10.1371/journal.pone.0045331

**Published:** 2012-09-18

**Authors:** Mian Xie, Ming Liu, Chao-Sheng He

**Affiliations:** 1 China State Key Laboratory of Respiratory Disease, The First Affiliated Hospital of Guangzhou Medical University, Guangzhou, China; 2 Guangzhou Institute of Respiratory Disease, The First Affiliated Hospital of Guangzhou Medical University, Guangzhou, China; 3 Department of Internal Medicine, Guangdong Provincial People’s Hospital, Guangzhou, China; University of Birmingham, United Kingdom

## Abstract

**Background:**

Sirtuin 1 (SIRT1) acts as a key regulator of vascular endothelial homeostasis, angiogenesis, and endothelial dysfunction. However, the underlying mechanism for SIRT1-mediated lung carcinoma angiogenesis remains unknown. Herein, we report that the nicotinamide adenine dinucleotide 1 (NAD1)-dependent deacetylase SIRT1 can function as an intrinsic negative modulator of Delta-like ligand 4 (DLL4)/Notch signaling in Lewis lung carcinoma (LLC) xenograft-derived vascular endothelial cells (lung cancer-derived ECs).

**Principal Findings:**

SIRT1 negatively regulates Notch1 intracellular domain (N1IC) and Notch1 target genes HEY1 and HEY2 in response to Delta-like ligand 4 (DLL4) stimulation. Furthermore, SIRT1 deacetylated and repressed N1IC expression. Quantitative chromatin immunoprecipitation (qChIP) analysis and gene reporter assay demonstrated that SIRT1 bound to one highly conserved region, which was located at approximately −500 bp upstream of the transcriptional start site of Notch1,and repressed Notch1 transcription. Inhibition of endothelial cell growth and sprouting angiogenesis by DLL4/Notch signaling was enhanced in SIRT1-silenced lung cancer-derived EC and rescued by Notch inhibitor DAPT. In vivo, an increase in proangiogenic activity was observed in Matrigel plugs from endothelial-specific SIRT1 knock-in mice. SIRT1 also enhanced tumor neovascularization and tumor growth of LLC xenografts.

**Conclusions:**

Our results show that SIRT1 facilitates endothelial cell branching and proliferation to increase vessel density and promote lung tumor growth through down-regulation of DLL4/Notch signaling and deacetylation of N1IC. Thus, targeting SIRT1 activity or/and gene expression may represent a novel mechanism in the treatment of lung cancer.

## Introduction

The nicotinamide adenine dinucleotide (NAD)-dependent deacetylase Sir2 regulates life span in multiple organisms in response to caloric restriction [1]. Mammalian homologues of Sir2 comprise a family of seven proteins, which are referred to as sirtuins (the class III histone deacetylases (HDAC) SIRT1-SIRT7), and have been characterized as key regulators of several important physiological processes associated with metabolism and stress resistance [2].

Recent studies have suggested that SIRT1 is an important mediator of vascular endothelial homeostasis, specifically contributing to angiogenesis, vascular tone, and endothelial functions [3]. Notably, SIRT1 is highly expressed in the vasculature during blood vessel growth, where it controls the angiogenic activity of endothelial cells. Additionally, loss of SIRT1 results in the down-regulation of genes related to blood vessel development and vascular remodeling, which leads to the inhibition of sprouting angiogenesis and the branching morphogenesis of endothelial cells [4].

The Notch pathway regulates numerous cell fate/lineage decisions in multicellular organisms during normal embryogenesis and postnatal development [5]. Upon binding of the Notch ligand to its cognate receptor, a proteolytic cleavage cascade is initiated in the intramembrane domain of the receptor. The final cleavage step is catalyzed by the γ-secretase protein complex and leads to the release of the active Notch intracellular domain. This protein fragment then translocates into the nucleus and functions as a cofactor to regulate the transcription of Notch target genes [6]. In addition to the Notch1 and Notch4 receptors, vascular endothelial cells also express Jagged 1, Delta-like ligand 1 (DLL1), and the endothelial cell-specific DLL4 [7]. Zhang et al. reported that the inactivation of a single allele of DLL4 led to embryonic lethality, suggesting that DLL4/Notch signaling is essential for vascular development. DLL4 expression is largely restricted to the endothelium of developing vessels and the tip cells of developing arteries, where it functions to regulate the number of tip cells to control vessel sprouting and branching [8].

Numerous studies have suggested that alterations in Notch activity can have profound effects on endothelial behavior and blood vessel formation. However, little is known about the regulation and adaptation of endothelial Notch responses in lung cancer angiogenesis. In the current study, we isolated vascular endothelial cells from Lewis lung carcinoma (LLC) subcutaneous xenografts in endothelial cell-specific SIRT1 knock-in C57BL/6J mice. Our results show that endothelial cells lacking SIRT1 activity are sensitized to Notch signaling, resulting in enhanced Notch target gene expression and impaired sprout elongation in response to DLL4 stimulation. Our data support the hypothesis that Notch signaling is dynamically regulated by acetylation and suggest that SIRT1 acts as a rheostat to fine-tune endothelial Notch responses.

## Results

### Isolation of Lung Cancer Xenograft-derived Vascular Endothelial Cells (Lung Cancer-Derived ECs)

**Figure 1:**
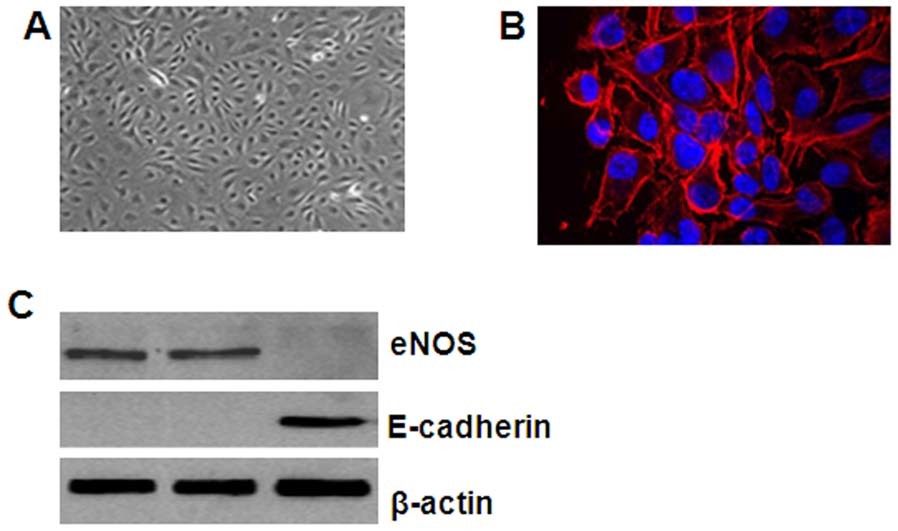
Isolation of lung cancer xenograft-derived ECs. LLC xenografts were resected from mice injected subcutaneously at the dorsal flank with LLC cells (3×10^6^ suspended in 50 µL PBS) for 30 days. After removing obvious necrotic tissues or extra fatty compositions, the minced tissues were ground on ice using a glass grinder and were then filtered through cell strainers to eliminate tissue debris. (A) The CD31-expressing lung cancer-derived ECs were isolated from the single-cell suspension by immunomagnetic sorting, as evidenced by scanning microscopy, and cultured in vitro. (B) CD31 was detected in the isolated lung cancer-derived ECs using immunofluorescence. CD31 antibody staining of the lung cancer-derived EC membranes is shown in red, and nuclear DAPI staining is shown in blue. (C) Total protein was isolated from enriched lung cancer-derived ECs, bEnd.3 cells (positive control), and MLE-12 (negative control) cells. Western blot analysis was performed using antibodies against eNOS and E-cadherin, and β-actin was used as the internal control.

Lung cancer-derived ECs were isolated from LLC subcutaneous xenografts in endothelial cell-specific SIRT1 knock-in C57BL/6J mice [9] (Fig. 1A). As shown in Fig. 1B, the lung cancer-derived ECs demonstrated typical cobblestone morphology and expressed CD31, a maker of mature endothelial cells. Moreover, these ECs expressed high levels of EC-specific endothelial nitric oxide synthase (eNOS) but not E-cadherin (Fig. 1C). These data suggest that the LLC xenogaft was efficiently enriched in lung cancer-derived ECs. These cells were then used for the subsequent in vitro experiments.

### Effect of Hypoxia on SIRT1 Expression in Lung Cancer-derived ECs

**Figure 2:**
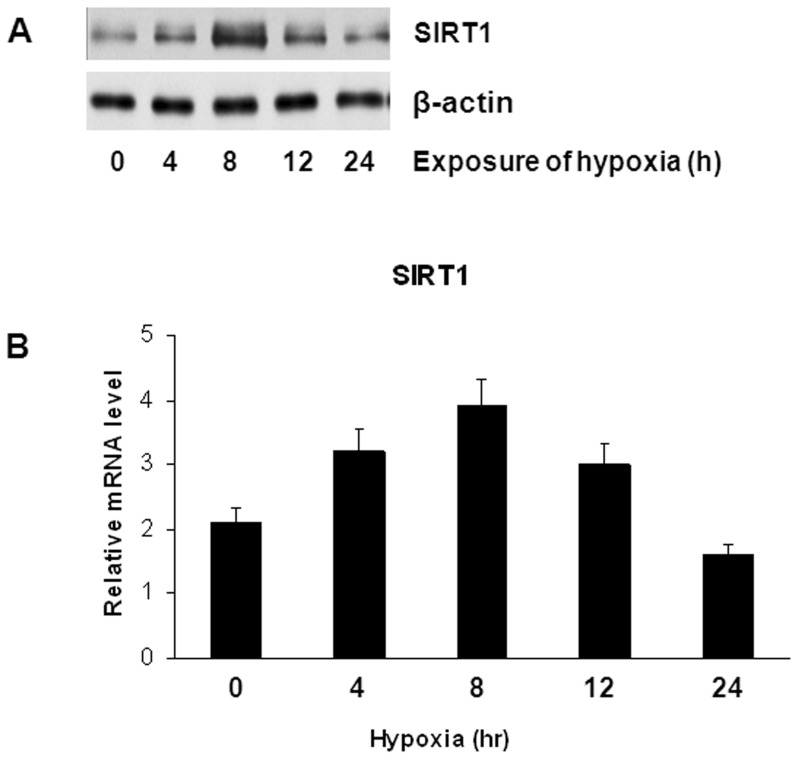
Hypoxia increases SIRT1 expression in lung cancer-derived ECs. (A) Western blot analysis of SIRT1 in lung cancer-derived ECs exposed to hypoxia (2% O_2_) for the indicated time using an antibody against SIRT1. (B) SIRT1 mRNA levels, as measured by real-time RT-PCR, of total RNA obtained from lung cancer-derived ECs exposed to hypoxia (2% O_2_) for the indicated time period. The data represent the average of three independent experiments from each time point performed in triplicate. * P<0.05 as compared to the control.

SIRT1 expression was assessed in the lung cancer-derived ECs, and as shown in Fig. S2A, SIRT1 was highly expressed in these cells. Because hypoxia is a feature of most tumors, we examined SIRT1 expression under different hypoxic conditions in these cells using western blot analysis. Our results indicated that the SIRT1 protein levels were increased in a time-dependent manner and reached the maximal at 8 h after exposure the lung cancer-derived ECs to hypoxia (Fig. 2A). To determine whether these changes in the SIRT1 protein levels during hypoxia were due to changes in SIRT1 gene transcription, we measured SIRT1 mRNA levels using real-time RT-PCR. Our data revealed the similar findings as observed in the SIRT1 protein expression (Fig. 2B). These results demonstrated the effect of hypoxia on SIRT1 expression in lung cancer-derived ECs.

### SIRT1 Negatively Regulates N1IC by Deacetylation and Controls Notch Target Genes in Lung Cancer-derived ECs

**Figure 3:**
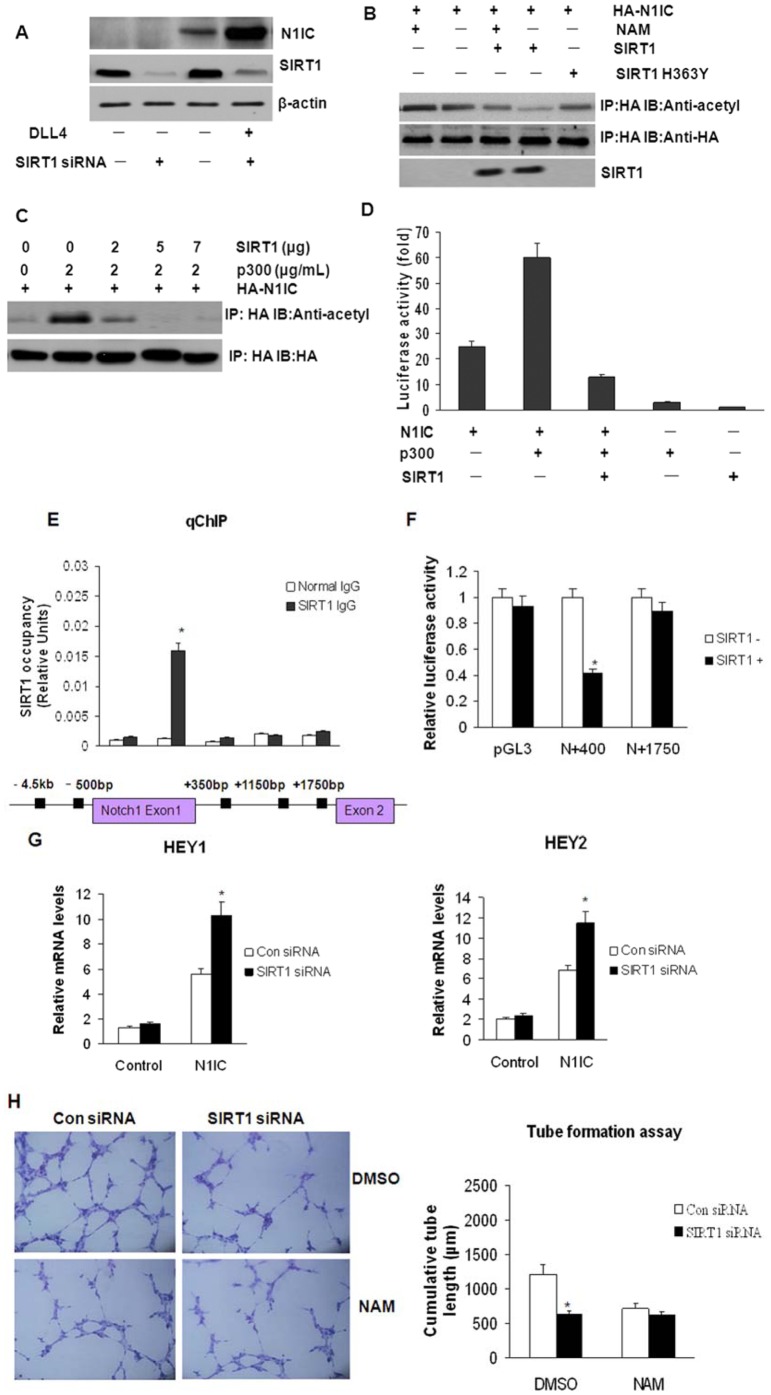
SIRT1 negatively regulates N1IC by deacytelation and regulates angiogenic activity in lung cancer-derived ECs. (A) Lung cancer-derived ECs were transfected with control or SIRT1 siRNAs for 48 h and cultured in the absence or presence of DLL4 for an additional 6 h. Then, N1IC protein expression was detected using western blot analysis. siRNA-mediated blockade of SIRT1 activity in endothelial cells increased the endogenous N1IC protein levels. (B) Lung cancer-derived ECs were transfected with HA-N1IC, pcDNA-SIRT1, pcDNA-SIRT1 H363Y, or pcDNA3 (4 µg/µL each) for 48 h and were then treated with or without 5 nM NAM for an additional 12 h. Afterward, the cell extracts were immunoprecipitated with an anti-HA antibody and subjected to western blot analysis with an anti-acetyl lysine (upper panel) or anti-HA antibody (lower panel). (C) Lung cancer-derived ECs were triple transfected with N1IC, the acetyltransferase p300, and increasing amounts of SIRT1, and the cells were lysed 48 h later. Comparable levels of N1IC were immunoprecipitated and blotted for acetylated lysine residues (IP: HA and IB: anti-acetyl; upper panel). The blot was reprobed for HA, which demonstrated approximately equal levels of HA-N1IC (IP: HA and IB: HA; lower panel). (D) Lung cancer-derived ECs were transfected with N1IC, the acetyltransferase p300, and increasing amounts of SIRT1 together with the Renilla luciferase reporter and the pGL3-CBF plasmid containing a firefly luciferase reporter gene for 24 h. Then, luciferase activity was measured using a dual-luciferase reporter assay. The data were normalized to the Renilla luciferase activity (mean ± SD; n = 3). (E) qChIP analysis of SIRT1 occupancy at the Notch1 proximal promoter region in lung cancer-derived ECs. SIRT1 occupied a specific region at −500 bp of the Notch1 locus. SIRT1 was immunoprecipitated (IP) with anti-SIRT1 sera (SIRT1 IgG) or preimmune sera (normal IgG, used as a control) (mean ± SEM; n = 3). * P<0.05 as compared to the control. (F) Luciferase reporter gene assay results. pGL3, pGL3-Notch1 −500 to +400 (N+400), or pGL3-Notch1+400 to +1750 (N+1750) were transfected into HEK293 cells with or without SIRT1. *P<0.05. The error bars represent the SEM. (G) Expression of the Notch target genes HEY1 and HEY2 were assessed in control siRNA- and SIRT1 siRNA-transfected lung cancer-derived ECs, which were subsequently transfected with empty vector or N1IC for up to 48 h. SIRT1-deficient endothelial cells displayed markedly enhanced HEY1 and HEY2 activity in response to N1IC overexpression. *P<0.05 as compared to the control. (H) Lung cancer-derived ECs were transfected with control or SIRT1 siRNAs and treated with DMSO or 20 mM NAM for 24 h, followed by incubation with Matrigel for an additional 5 h. Next, tube formation was evaluated using an inverted phase microscope. The bar graphs represent the densitometry results. * P<0.05 as compared to the control.

Upon translocation to the nucleus, the intracellular domain of Notch1 (N1IC) interacts with the DNA-binding CSL protein (named for CBF1 in mammals, Su (H) in Drosophila, and Lag-1 in Caenorhabditis elegans), which results in the transcriptional activation of Notch1 gene targets. Because N1IC undergoes rapid ubiquitin-mediated degradation and acetylation can impair ubiquitination [10], we examined the effect of SIRT1 on N1IC expression and found that inactivation of SIRT1 led to increased N1IC protein expression in response to DLL4 stimulation (Fig. 3A). Taken together, these results indicated that SIRT1 negatively regulates Notch signaling in endothelial cells when DLL4/Notch1 signals are activated.

We next tested whether SIRT1 negatively regulates N1IC via deacetylation. Briefly, different versions of HA-tagged N1IC (HA-N1IC) were expressed in lung cancer-derived ECs with vectors encoding either SIRT1 or a SIRT1 mutant lacking deacetylase activity (SIRT1 H363Y). Cell extracts were immunoprecipitated with an anti-HA antibody and detected with an immunoblot using an antibody against acetyl-lysine. Our results demonstrated that the expression of acetylated HA-N1IC was significantly reduced in cells expressing full SIRT1, as compared to the protein levels in cells expressing SIRT1 H363Y (Fig. 3B). However, when the cells were treated with 5 nm NAM, the SIRT1-mediated inhibition of N1IC acetylation was relieved. Furthermore, transfection of lung cancer-derived ECs with N1IC, the acetyltransferase p300, and increasing amounts of SIRT1 revealed that p300 was capable of acetylating N1IC (Fig. 3C) and enhancing N1IC transcriptional activity (Fig. 3D). However, the addition of SIRT1 abolished the acetylated form of N1IC and significantly diminished N1IC activity induced by p300. These results suggested that the deacetylases activity of SIRT1 reduced the abundance of acetylated N1IC. Moreover, activation of SIRT1 by SRT1720 or SRT2183 (activators of the NAD^+^-dependent deacetylase) reduced endogenous N1IC protein levels (Fig. S1A), and blocking the deacetylase activity of SIRT1 with the sirtuin inhibitor nicotinamide (NAM) increased endogenous N1IC levels in endothelial cells (Fig. S1B).

To define the mechanism by which SIRT1 represses N1IC expression, we examined the recruitment of SIRT1 to the Notch1 locus in response to DLL4 stimulation using qChIP analysis. Of note, the Notch1 locus is thought to contain more than 20 highly conserved regions [11]. Our data indicated that SIRT1 bound to one highly conserved region, which was located at approximately −500 bp upstream of the transcriptional start site of Notch1 (Fig. 3E). This finding suggests that SIRT1 may directly control Notch1 expression through binding to the proximal promoter region of Notch1. To determine the functional consequence of SIRT1 binding to the Notch1 locus, luciferase reporter constructs containing Notch1 reporter gene were transfected into HEK293 cells. These results demonstrated that SIRT1 significantly inhibited Notch1 reporter activity when the SIRT1 binding site was present (Fig. 3F, N+400), which was not observed when the SIRT1 binding site was absent (Fig. 3F, N+1750). This further confirmed our observation that SIRT1 repressed Notch1 transcription through binding to the Notch1 promoter.

HEY1 and HEY2 are direct target genes of the Notch signaling pathway during vascular development [12]. To further investigate the effect of SIRT1 on HEY expression, SIRT1 expression was silenced in lung cancer-derived ECs by siRNA, and the expression of HEY1 and HEY2 was determined. Notably, SIRT1-deficient lung cancer-derived ECs overexpressing N1IC displayed a markedly enhanced level of HEY1 and HEY2 activity (Fig. 3G). Similarly, the silencing of SIRT1 significantly enhanced HEY1 and HEY2 expression in lung cancer-derived ECs in response to DLL4 stimulation, whereas no changes were observed in unstimulated lung cancer-derived ECs (Fig. S1C). This result indicated that SIRT1 regulation of Notch target genes was dependent on DLL4 stimulation. Furthermore, the enhanced Notch responsiveness to DLL4 of the SIRT1-deficient lung cancer-derived ECs was abrogated by the addition of the γ-secretase inhibitor DAPT (Fig. S1C). In contrast, activation of SIRT1 signaling through the use of the small molecules SRT1720 or SRT2183 inhibited DLL4-mediated induction of HEY1 and HEY2 expression, indicating that SIRT1 negatively modulated DLL4/Notch1 signaling in lung cancer-derived ECs (Fig. S1D).

### SIRT1 Regulates the Angiogenic Activity of Lung Cancer-derived ECs

The inhibition of endothelial cell growth by DLL4/Notch signaling was enhanced in SIRT1-silenced lung cancer-derived ECs and was rescued by Notch inhibitor DAPT (Fig. S2A). In the late stage of angiogenesis, endothelial cells self-assemble into tubes to form new blood vessels [13]. We then investigated the effects of SIRT1 on neovascularization by examining tube formation. As shown in Fig. 3H, tubular formation was significantly inhibited in SIRT1-silenced lung cancer-derived ECs. Similarly, tube formation in lung cancer-derived ECs was also repressed when the cells were treated with the SIRT1 deacetylase inhibitor NAM. To confirm this SIRT-mediated regulation of endothelial cell function, three-dimensional in vitro angiogenesis assays were performed using collagen gel-embedded endothelial spheroids that had been transfected with control or SIRT1 siRNAs. As shown in Fig. S2B, the cumulative sprout lengths of spheroids in lung cancer-derived ECs transfected with SIRT1 siRNA were significantly shorter than that in the control-transfected cells. Moreover, treatment with DAPT abrogated the inhibition of the angiogenic response to lung cancer-derived ECs induced through SIRT1 silencing, suggesting that SIRT1 is an important modulator of endothelial angiogenic function.

### Effect of SIRT1 on Tumor Neovascularization in LLC Xenografts

**Figure 4:**
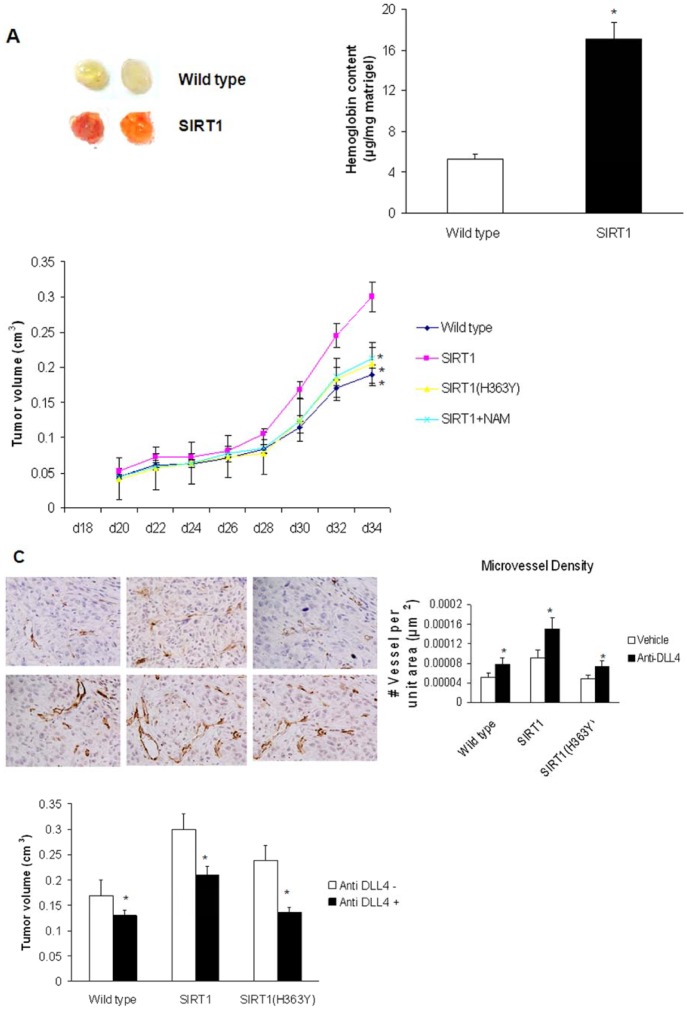
Effect of SIRT1 on tumor neovascularization in LLC xenografts. (A) SIRT1 promotes angiogenesis in vivo. Matrigel plugs were subcutaneously injected into the abdomens of control or SIRT1-transgenic mice, and the plugs were extracted 7 days later to determine the extent of vascularization. The amount of hemoglobin present in the plugs was quantified as an indicator of the formation of functional blood vessels (mean ± SD; n = 10 for each group). * P<0.05 as compared to the control. (B) Changes in the median tumor volumes measured in wild-type (control) C57BL/6J, SIRT1, or SIRT1 (H363Y) mice following inoculation with LLC cells for the indicated time period. * P<0.05 as compared to the SIRT1 mice. (C) Photomicrographs of CD31 IHC staining in sections of LLC xenografts (magnification, ×200) from wild-type, SIRT1 or SIRT1(H363Y) mice. When the xenograft tumor volumes reached approximately 100 mm^3^, the mice were randomly assigned to the control arm (n = 8) or the experimental arm (DLL4 monoclonal antibody; n = 10). The high-magnification fields (×400) were analyzed for microvessel density count using ImageJ software (mean ± SD; n = 10 for each group). *P<0.05 as compared to the SIRT1 mice. The average tumor volumes in the wild-type, SIRT1 and SIRT1 (H363Y) mice were measured at 4 weeks after treatment. * P<0.05 as compared to the control.

To investigate the role of SIRT1 on vascularization in vivo, a Matrigel plug assay was performed. In contrast to the plugs in control C57BL/6J mice, the plugs in SIRT1 knock-in transgenic mice displayed a bright red coloration, which indicated that SIRT1 had induced new blood vessel formation. The hemoglobin level, which is indicative of the extent of angiogenesis, was higher in the Matrigel plugs from SIRT1 transgenic mice than those from control C57BL/6J mice (Fig. 4A). To investigate the effect of the SIRT1 gene on tumor vascularization and tumor growth, LLC cells were injected subcutaneously into SIRT1, SIRT1 H363Y, and wild-type C57BL/6J mice. These results indicated that LLC tumor volume in the SIRT1 knock-in transgenic mice was 115% of that observed in the control mice 30 days after tumor cell implantation. In contrast, tumor growth in the SIRT1 H363Y transgenic mice was reduced and was similar to that observed in the wild-type mice. Furthermore, in the SIRT1 transgenic mice, tumor growth was decreased when the deacetylase activity of SIRT1 was blocked with the administration of the class III HDAC inhibitor nicotinamide (Fig. 4B). Upon assessment of the microvessel density index by CD31 antibody staining, the tumor growth enhancement observed in the SIRT1 knock-in transgenic mice was associated with an increase in tumor vascularization. In addition, anti-Dll4 treatment revealed that the reduced tumor growth was associated with an apparent decrease in tumor vascular density (Fig. 4C).

## Discussion

This study expands the role of SIRT1 to include the specific modulation of angiogenic activity in endothelial cells. To examine the effect of SIRT1 on lung cancer angiogenesis, we established LLC xenograft models with endothelial cell-specific SIRT1 and SIRT1 H363Y expression in transgenic mice. Endothelial cells were successfully isolated from the LLC xenografts of SIRT1 transgenic mice using CD31-conjugated magnetic beads as well as a series of steps to remove all contaminating tumor cells and leukocytes. Blockade of SIRT1 function abolished endothelial tube formation and sprout formation in vitro, which was abrogated by Notch inhibitor. These results suggest that SIRT1-mediated regulation of the Notch pathway in endothelial cells can be modulated.

In our studies, SIRT1 was highly expressed in lung cancer-derived ECs. Moreover, hypoxia has been shown to be one of the major triggers for new blood vessel growth in malignant tumors [14]. Zhang et al. demonstrated that because HIC1 (Hypermethylated in Cancer 1) bonded to the SIRT1 promoter, the consequent reduction of CtBP recruitment decreased transcriptional repression and induced SIRT1 expression in lung cancer cells [15]. We hypothesized that SIRT1 regulation during hypoxia could be conferred via changes in SIRT1 gene expression in lung cancer-derived ECs. To our knowledge, this was the first study to demonstrate that SIRT1 expression is increased in a time-dependent manner during hypoxia in tumor endothelial cells.

We next asked whether SIRT1 interacted with particular transcriptional partners in vascular endothelial cells to mediate its specific effects. Guarani et al. [16] previously showed that acetylation of N1IC on conserved lysines controlled the amplitude and duration of Notch responses through altered N1IC protein turnover. SIRT1 associates with N1IC and functions as a N1IC deacetylase to inhibit acetylation-induced N1IC stabilization. Consistently, our results also indicated that SIRT1 deacetylated and repressed N1IC expression, which led to enhanced angiogenic sprouting and tubulogenesis regulated by the SIRT1-dependent angiogenic signaling pathway. More importantly, we showed for the first time that SIRT1 was recruited to one highly conserved region located at −500 bp upstream of the Notch1 transcriptional start site, which suggested that SIRT1 could directly bind to the Notch1 promoter, thereby inhibiting gene expression. Of notes, SIRT1 negatively regulated Notch1 via deacetylation, thus changes at transcriptional level of Notch may also play a role in the reduction of Notch activity affected by SIRT1. This needs to be further determined.

HEY is helix-loop-helix (bHLH) transcriptional repressor. HEY family members are considered target genes of Notch1 signaling in the endothelium because their expression can be induced by Notch1 [12]. In this study, we examined the ability of SIRT1 to regulate the expression of genes controlled by N1IC, such as HEY1 and HEY2. In agreement with reports by Mulligan et al. [17], our results revealed that SIRT1 negatively regulated the expression of HEY1 and HEY2 in lung cancer-derived ECs. Moreover, Takara et al. demonstrated that SIRT1 functionally interacts with the Hairy and enhancer-of-split bHLH repressors [18]. To further investigate the effects of SIRT1 on the Notch-mediated cell response and tumor growth, we investigated sprout formation in lung cancer-derived ECs and found that inactivation of SIRT1 impaired sprout formation and elongation. These effects were reversed by the addition o the Notch1 inhibitor DAPT, suggesting that SIRT1 modulates endothelial angiogenic functions by regulating Notch signaling. However, the inactivation of SIRT1 did not completely prevent sprout formation. Thus, we hypothesized that, in addition to Notch1, novel SIRT1-regulated transcription factors may also be involved in vascular development, although future studies are required to address the role of these unknown regulators in lung cancer.

Guarani et al. reported that p300 could bind to N1IC and function as a co-activator at Notch-regulated promoters [16]. Moreover, our study showed that p300 was not only capable of acetylating N1IC but could also activate its transcription. More importantly, SIRT1 inhibited the acetylated form of N1IC and significantly diminished N1IC activity in the presence of p300. Conversely, treatment with the deacetylase inhibitor NAM led to significant increases in N1IC mRNA expression. Thus, we concluded that SIRT1 deacetylase activity altered the endothelial Notch signaling cascade by deacetylating N1IC and antagonizing p300. Hansson et al. showed that MAML1 (Notch coactivator) could potentiate p300 autoacetylation and p300 transcriptional activation [19]. However, our results suggest that SIRT1 physically interacts with and represses p300 transactivation in an NAD-dependent manner. SIRT1 repression of p300 has been shown to occur via deacetylation of two lysine residues (1020 and 1024) within the p300 cysteine-rich domain (CRD1) [20]. Because p300 is a gating transcriptional cofactor, deacetylation and repression of p300 by SIRT1 may serve as an important integration point during metabolism and cellular differentiation.

Upon the binding of a transmembrane ligand from the Delta/Jagged families, Notch transmembrane receptors generally provide signals that guide cell-fate decisions. In particular, DLL4 is required for vascular development and is strongly expressed in tumor vessels [21]. In addition, the study of SIRT1 knock-in mice indicated that SIRT1 functioned to maintain neovascularization capacity in response to DLL4 stimulation in the tumor vascular endothelium. To determine the function of DLL4 during tumor angiogenesis in the SIRT1 transgenic mice, we decreased the DLL4 level present in a murine tumor model using an anti-DLL4 monoclonal antibody. Our results suggest that the inhibition of DLL4-Notch signaling resulted in an increased vascular tumor density. Surprisingly, this vascular overgrowth phenotype resulted in the inhibition of tumor growth. One plausible explanation could be that excessive branching results in a highly chaotic vascular network lacking the hierarchy essential for efficient directional blood flow. Perfusion studies demonstrated that hypersprouting tumor vasculature was non-functional [6], which suggested that blockade of DLL4-Notch signaling could lead to tumor vessel “abnormalization” and inhibited tumor growth. In endothelial cells, SIRT1 modulates the transcriptional activity of Foxo1 and p53, and activates the enzymatic activity of endothelial nitric oxide synthase (eNOS). Given the multitude of downstream factors targeted by SIRT1 for deacetylation, SIRT1 has been hypothesized to act as a key regulator of several pathways involved in tumor growth and vascularization [22]. Our findings suggest that pharmacological- or molecular-based blockade of SIRT1 activity may represent a promising and novel approach for blocking tumor progression.

**Figure 5:**
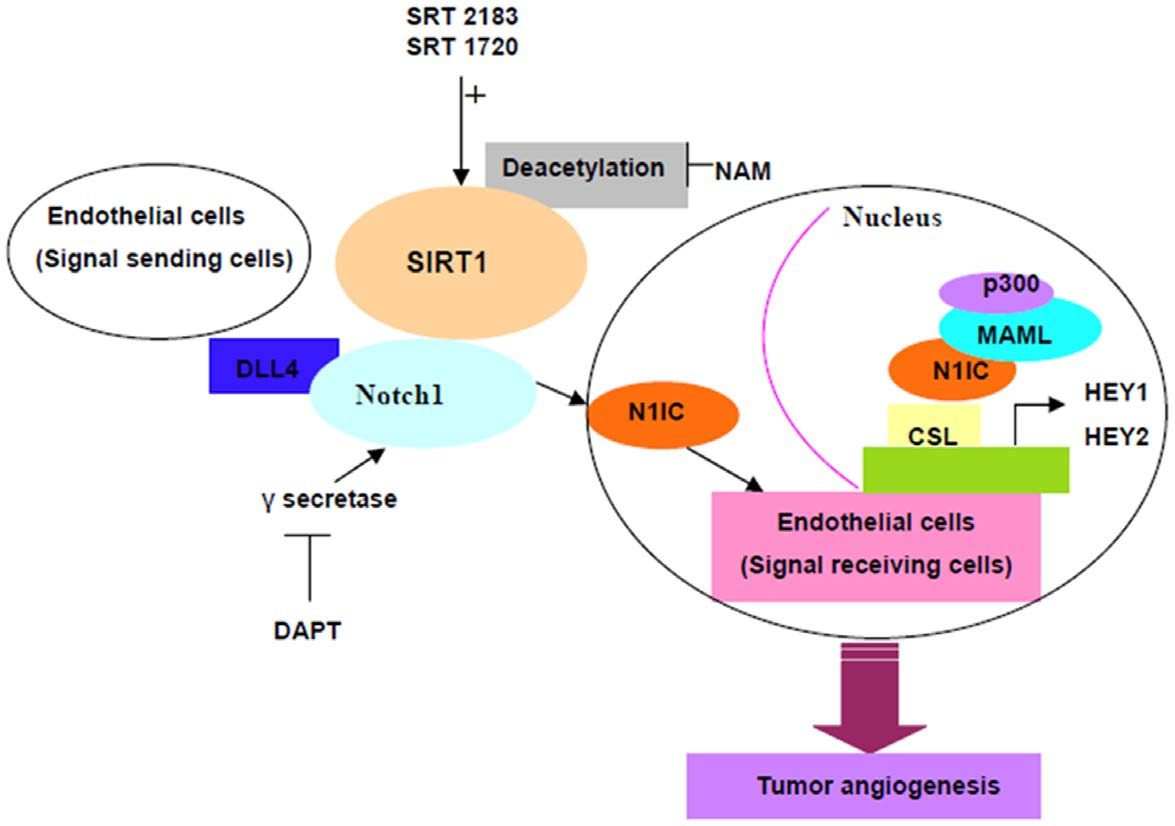
A model for SIRT1-mediated regulation of endothelial DLL4/Notch signaling in lung cancer angiogenesis. Our study show that the Delta-like ligand 4 (DLL4), which is highly expressed in vascular cells during tumor angiogenesis, binds to the Notch1 receptor and initiates proteolytic cleavages. The final intramembrane cleavage catalyzed by γ-secretase leads to the release of the active Notch1 intracellular domain (N1IC), which translocates into the nucleus and recruits the protein MAML1 and histone acetyltransferases (HATs, such as p300) to the CSL complex. This recruitment leads to the activation of the Notch1 target genes HEY1 and HEY2. Moreover, p300 acetylates N1IC and enhances its transcriptional activity whereas SIRT1 inhibits the acetylated form of N1IC and significantly diminishes N1IC activity induced by p300, thereby limiting the DLL4/Notch signaling response and inhibiting tumor angiogenesis.

Collectively, our study demonstrate a novel role for SIRT1, whereby SIRT1 acts as a cell-autonomous negative modulator of Notch signaling, and these results identify the reversible acetylation of N1IC as a key molecular mechanism by which Notch responses can be dynamically modulated. SIRT1 inhibits the acetylated form of N1IC and significantly diminishes N1IC activity induced by p300, thereby limiting the DLL4/Notch signaling response and inhibiting tumor angiogenesis (Fig. 5). Our results suggest that blockade of SIRT1 activity or/and gene expression may provide novel opportunities for anti-cancer treatment.

## Materials and Methods

### Ethics Statement

All animal work was conducted under the institutional guidelines of Guangdong Province and approved by the Use Committee for Animal Care. Approval was also obtained from the ethics committee of Guangzhou Institute of Respiratory Disease Research (ID. 2009–2130).

### Reagents and *in vitro* Cell Treatments

Nicotinamide and N-[N-(3, 5-difluorophenacetyl)-L-alanyl]-S- phenylglycine t-butyl ester (DAPT) were obtained from Sigma-Aldrich (USA). SRT2183 and SRT1720 were purchased from Sirtris Pharmaceuticals (USA). DLL4 was purchased from R&D Systems (USA). Control groups were treated with the respective vehicles. The anti-DLL4 monoclonal antibody (a human IgG1 isotype that cross-reacts with both human and mouse DLL4, 1 µg/mL) was a gift from MedImmune, LLC (USA).

### Xenograft Models

C57BL/6J mice (females, 6–8 weeks old, weighing 15–22 g) were purchased from the Shanghai Slac Laboratory Animal Co., Ltd. (China). Endothelial cell-specific SIRT1-knock-in or SIRT1 (H363Y)-transgenic C57BL/6J mice were provided by the Chinese Academy of Medical Sciences. To generate endothelium-specific SIRT1 or SIRT1 (H363Y)-transgenic mice, human SIRT1 cDNA (NCBI ref: NM_012238.4) or SIRT1 (H363Y) cDNA was ligated with the mouse VE-Cadherin promoter, and the resulting clones were referred to as VE-S. Endothelial cell-specific transgenic mouse lines were established by microinjecting the respective VE-S plasmids into fertilized C57BL/6J eggs [9]. Wild-type C57BL/6J (control) mice, SIRT1, and SIRT1 (H363Y)-transgenic mice were injected subcutaneously at the dorsal flank with LLC cells (3×10^6^ cells suspended in 50 µL PBS). The SIRT1-transgenic mice were divided into two groups: one group was orally administered sterile physiological saline solution (0.1 mL), whereas the other group was orally administered the sirtuin inhibitor nicotinamide (5 µmol/mouse) dissolved in sterile saline (0.1 mL). Mouse body weight and tumor size were measured at different time points following tumor implantation, and the values were applied to the following formula: [1/2 (Length×Width^2^)]. On day 34, the mice were scarified, and the xenografted tumors were removed.

For the LLC xenograft experiments to investigate the influence of DLL4 on tumor angiogenesis in wild-type C57BL/6J mice, endothelial cell-specific SIRT1, and SIRT1 (H363Y)-transgenic mice were randomly assigned to the control arm (n = 9) or the experimental arm (DLL4 monoclonal antibody; n = 12) when the xenograft tumor volume reached approximately 100 mm^3^. Mice then received intraperitoneal injections of 100 µL PBS (control arm) or 10 mg/kg DLL4 monoclonal antibody once per week (anti-DLL4 treatment arm) for up to 4 weeks.

### Isolation of Lung Cancer Xenograft-derived Vascular Endothelial Cells

LLC xenografts from the SIRT1 transgenic mice were immersed in ice-cold phosphate buffered saline (PBS) immediately after tumor resection. The collected tissues (1 g) were placed on ice and minced into fine pieces using a surgical blade, after which a single wash of 0.1% bovine serum albumin (BSA)/PBS was applied to remove excrescent tissues. After removing obvious necrotic tissues or extra fatty compositions, the minced tissues were ground on ice using a glass grinder and then filtered through cell strainers to eliminate tissue debris. Filtered cells were then washed via resuspension and centrifugation at 235 g for 5 min at 4°C. The cell pellets were then resuspended in 0.1% BSA/PBS. Next, CD31 immunomagnetic beads (Dynal Biotech, Norway) were gently added to the cell pellet, and the mixture was incubated with gentle tilting and rotation for 40 min at 4°C. Afterward, the CD31-expressing lung cancer-derived ECs complexed to beads were isolated and washed via the application of magnetic force. The lung cancer-derived ECs were eluted, and CD31 expression was detected using a CD31 monoclonal antibody (Cell Signaling Technology, USA) and staining with NorthernLights 557-conjugated anti-mouse IgG secondary antibody. 4′,6-diamidino-2-phenylindole (DAPI, Sigma-Aldrich) counterstaining was used to detect the nucleus [23].

### Immunohistochemistry (IHC)

Cryostat sections of frozen tumors were fixed with 4% paraformaldehyde and washed with PBS, and the endogenous peroxidase activity was blocked with a dual-endogenous enzyme blocking solution (Dako, Denmark). The sections were blocked with 5% fetal bovine serum (FBS; Gibco, USA) in PBS. To detect CD31-positive microvessels, the sections were probed with a CD31 monoclonal antibody (Cell Signaling Technology) and were then incubated with a horseradish peroxidase (HRP)-conjugated secondary antibody using the Rat on Mouse HRP-Polymer Kit (Biocare Medical, USA). Following color development using the diaminobenzidine (DAB) reagent (Dako), the nuclei were stained with hematoxyline. The sections were then sealed with glycerol-gelatin (Sigma-Aldrich) for microscopic observation. Randomly selected fields were photographed at ×200 magnification, and the numbers of CD31-positive blood vessels were counted.

The two most vascularized areas were imaged at a low magnification (×40), and the vessels in these two areas were counted in a representative high magnification field (×400; 0.152 mm^2^; 0.44 mm diameter) using ImageJ software. Mean visual microvessel density for CD31 was calculated as the average of four counts. Values are expressed as the means ± standard deviation (SD).

### Endothelial Cell Proliferation Assay

Cell growth was examined using a colorimetric procedure with the Cell Counting Kit-8 according to the instruction from the manufacturer (Dojindo Laboratories, Japan). Briefly, lung cancer-derived ECs were treated with DLL4 (500 µg/mL) for 1 h followed by exposing to DAPT (10 µM) for up to 72 h. Afterwards, the Cell Counting Kit-8 reagent was added to the cultures and cell growth was evaluated by measuring absorbance using microplate reader at 450 nm.

### Matrigel Plug Assay

Wild-type and SIRT1-transgenic mice were subcutaneously injected in the abdomen with 500 µL of growth factor-reduced Matrigel (BD Biosciences, USA) containing 200 ng FGF2 (R&D Systems) and 60 U/mL heparin (Sigma-Aldrich). Seven days later, the mice were scarified, and the Matrigel plugs were removed. The hemoglobin level in each plug was measured using Drakin’s Reagent Kit (Sigma-Aldrich) to quantitatively assess the formation of functional blood vessels [13].

### Tube Formation Assay

Matrigel was added to 24-well plates and allowed to solidify for 30 min at 37°C, after which point the lung cancer-derived ECs were seeded (5×10^4^ cells/well) on the solidified Matrigel. After incubation for 5 h, the cells were fixed with 4% paraformaldehyde and stained with 0.1% crystal violet in 20% methanol.

### Cells and Cell Culture

Lung cancer-derived ECs were plated, grown, and maintained in endothelial cell medium (EGM-2 BulletKit; Lonza, USA) supplemented with the following factors: human epidermal growth factor, hydrocortisone, GA-1000 (Gentamicin, Amphotericin-B), fetal bovine serum, vascular endothelial growth factor, human fibroblast growth factor (basic), long R3 insulin-like growth factor-I, ascorbic acid, and heparin.

The bEnd.3 (mouse brain microvascular endothelial cells) and MLE-12 (mouse lung epithelial cells) cell lines were purchased from ATCC. The bEnd.3 cells were cultured in Dulbecco’s Modified Eagle’s medium (DMEM; Gibco) supplemented with 10% FBS, and the MLE-12 cells were cultured in RPMI 1640 medium (Gibco) containing 10% FBS. DMEM/F-12 was supplemented with 10% FBS, 1% HEPES, 1% insulin/transferrin/sodium selenite, 0.01% β-estradiol, and 0.01% hydrocortisone.

### Cell Cultures in Hypoxic Conditions

For experiments performed in a reduced O_2_ atmosphere, lung cancer-derived ECs were incubated in a temperature- and humidity-controlled C chamber (BioSpherix, USA) and an atmosphere containing 2% O_2_, 5% CO_2_, and 90% N_2_, as described previously [17].

### DLL4 Stimulation of Endothelial Cells

Recombinant human DLL4 (R&D Systems) was reconstituted at 200 µg/mL in sterile PBS. DLL4 (500 µg/mL in PBS) was then immobilized onto culture dishes via incubation for 1 h at room temperature. Lung cancer-derived ECs were stimulated with DLL4 for 6 h upon their addition to the DLL4-coated dishes [24].

### RNA Interference

Lung cancer-derived ECs were seeded onto 12-well culture plates at a density of 1.5×10^5^ cells/well and incubated for 16 h prior to treatment with SIRT1 siRNA (sc-40986; Santa Cruz Biotechnology, Inc., USA) or control siRNA (Dharmacon, USA). The next day, the cells were washed twice with PBS. Lipofectamine™ 2000 (Invitrogen, USA) was used for transfection according to the manufacturer’s instructions.

### Plasmids and Transfection

Mouse cDNA encoding the Notch1 intracellular domain was amplified using total RNA isolated from the mouse EL4 lymphoma cell line. The following gene-specific primers were used: 5′-ATG TTC TTT GTG GGC TGT GGG-3′ and 5′-TGG CAG TGA TGT TGG TAG GGC-3′. The pcDNA-HA-N1IC plasmid, which is a derivative of the mammalian cell expression vector pcDNA3-HA2 and contains the cDNA-encoded 1747–2531 amino acid sequence of the mouse Notch1 receptor [25] with an HA tag at the N-terminus, was chosen as the expression vector. To generate the SIRT1 expression vectors, wild-type SIRT1 and a deacetylase-defective mutant of SIRT1 (SIRT1 H363Y) were excised from retroviral vectors [26] and inserted into the BamH I and BstX I sites of pcDNA3-HA. The SIRT1 fragments were also subcloned into pSilencer-Neo (Ambion, USA). The p300 plasmid was purchased from Addgene (USA). Transient transfections of HEK293 cells were performed with the Lipofectamine™ 2000 transfection reagent. Cells were grown to 60–70% confluence and then transfected using TransPass V reagents (New England Biolabs, USA), as recommended by the manufacturer.

### Reporter Gene Assay

Lung cancer-derived ECs were cotransfected using the SuperFect transfection reagent (Qiagen, Germany) with the Renilla luciferase reporter plasmid (Promega, USA) and the pGL3-CBF plasmid containing the firefly luciferase reporter gene (kindly provided by Dr. Hai Yu, Shanghai Jiaotong University, China) [27]. Luciferase activity in the cell lysates was determined using the Dual-Luciferase® Reporter Assay Kit (Promega) [28]. A Notch reporter plasmid containing the SIRT1 interaction region, which was previously identified by ChIP, was constructed (Fig. 3F, N+400) by subcloning the sequence of the Notch1 locus from the −500 bp region of the transcriptional start site to the +400 bp region into the pGL3 basic vector (Promega). A reporter gene without the SIRT1 interaction region was constructed by subcloning a region from the +400 bp to the +1750 bp of the Notch1 promoter into the pGL3 basic vector (Fig. 3F, N+1750). HEK293 cells in a 24-well plate were transfected with the reporter gene (25 ng/µL) with or without 100 ng of pCMV2-HA-SIRT1 in addition to 10 ng of β-galactosidase-CMV vector using the Lipofectamine Plus reagent (Invitrogen). Luciferase assays were performed at 24 h post-transfection using the luciferase assay system (Promega), and all values were normalized to β-galactosidase activity. The assays were repeated at least three times in duplicate.

### RNA Analysis Using Real-time Quantitative PCR

Total RNA was extracted from lung cancer-derived ECs using TRIzol (Invitrogen) and reverse-transcribed using the High Capacity cDNA Reverse Transcription Kit (Applied Biosystems, Inc., USA). Expression levels of HEY1, HEY2, SIRT1 and N1IC were measured via real-time PCR using the Assay-on-Demand Taqman Gene Expression probes (Applied Biosystems). The glyceraldehyde 3-phosphate dehydrogenase (GAPDH) housekeeping gene was amplified to serve as the internal calibrator. The PCR reaction conditions were set according to the manufacturer’s recommendations, and the fluorescence signals were monitored after each PCR cycle using the Prism 7900 sequence detection system (Applied Biosystems). CT values representing the cycle number where fluorescence exceeded a fixed threshold were obtained and normalized with the corresponding GAPDH CT values.

### Immunoprecipitation

Cell extracts were incubated with 4 µg of antibody for 4 h at 4°C, and protein G-agarose beads were added for an additional 4 h incubation. The beads were washed four times, and the immunoprecipitates were heated in sodium dodecyl sulfate-polyacrylamide electrophoresis (SDS-PAGE) sample buffer. Following brief centrifugation, the supernatants were resolved on an 8% SDS gel for subsequent immunoblot analysis. Commercially available anti-HA antibodies (1∶2000; Sigma-Aldrich) were used for immunoprecipitation.

### Western Blot Analysis

Cell extracts or immunoprecipitates were separated using SDS-PAGE and transferred to nitrocellulose membranes. The membranes were incubated with a primary antibody, and immunoreactive bands were visualized with an enhanced chemiluminescent reagent (Pierce, USA). The antibodies used included anti-acetyl lysine (1∶1000; Upstate Biotechnologies, USA) and anti-HA (1∶2000; Sigma-Aldrich). Additional antibodies used for the immunoblot analyses included anti-N1IC (1∶1000; Abcam, USA), anti-eNOS (1∶2000), anti-E-cadherin (1∶2000), anti-SIRT1 (1∶1000), and anti-β-actin (1∶1000) (all from Cell Signaling Technology).

### Quantitative Chromatin Immunoprecipitation Analysis

qChIP experiments were performed as described previously [29]. Primers were designed to amplify 50–150-bp amplicons. The products were measured using SYBR green fluorescence (ABI Prism 7300 System and Power SYBR Green Master Mix; Applied Biosystems, Inc.). The amount of product was determined relative to a standard curve of input chromatin. All of the experiments were repeated at least three times. The antisera used consisted of rabbit preimmune sera or rabbit anti-mouse SIRT1 antisera (both from Cell Signaling Technology). For amplification of the Notch1 locus, the following primers were used: −4.5 kb forward, 5′-TTT AAA TGG CCC TGA GCA AGA-3′; −4.5 kb reverse, 5′-AAA TTG CCC AAA CCA GAG ACA-3′; −500 bp forward, 5′-GCA GCT TTC CTT TCC CAC AA-3′; −500 bp reverse, 5′-TTT GGC CAG AAT TTG CAT TTC-3′; +350 bp forward, 5′-GAG GAG GAC CTT TCT CTT TCC A-3′; +350 bp reverse, 5′-TTG GGC GCT ATG AGA AAA GTG-3′; +1150 bp forward, 5′-CTG GAG ATG CCT GCG AAC A-3′; +1150 bp reverse, 5′-AGA CCT GCC CCG CCT ACT-3′; +1750 bp forward, AAC CAA GCC TGA CCT CTC TCT TC-3′; and +1750 bp reverse, 5′-CAC TTG GCT GGG A GC ATC TC-3′.

### Spheroid-based Angiogenesis Assay

Endothelial cell spheroids of a defined cell number were generated as described previously [30]. Briefly, 12 h after transfection, lung cancer-derived ECs were suspended in culture medium containing 0.2% (wt/vol) carboxymethylcellulose (Sigma-Aldrich) and seeded in non-adherent round-bottom 96-well plates (Greiner Bio-One, USA). Under these conditions, all suspended cells contributed to the formation of a single spheroid per well of a defined size and cell number (400 cells/spheroid). Spheroids were generated overnight and embedded into collagen gels. The spheroid-containing gels were rapidly transferred onto 24-well plates and allowed to polymerize for 30 min. Then, 100 µL endothelial basal medium (EBM; Cambrex, USA) was layered over the gel. After 24 h, in vitro angiogenesis was quantified by measuring the cumulative length of the vascular sprouts using ImageJ software. In total, 10 spheroids were analyzed per experimental group and experiment.

### Statistical Analysis

Data processing was performed using the Statistical Package for the Social Sciences (SPSS v.15.0; USA). All intergroup differences were analyzed using the Mann- Whitney-Wilcoxon test. All results are presented as the mean ± standard error of the mean (SEM) or standard deviation (SD). P-values less than 0.05 were considered statistically significant.

## Supporting Information

Figure S1 **SIRT1 negatively regulates N1IC and controls Notch target gene expression in lung cancer-derived ECs.** HEY1 and HEY2 mRNA levels were measured by real-time RT-PCR using total RNA obtained from lung cancer-derived ECs. The data represent the average of three independent samples obtained at each time point, and each sample was measured in triplicate. Values were expressed as relative arbitrary units following normalization to β-actin mRNA expression. (A) Lung cancer-derived ECs were cultured in either the absence or presence of DLL4 and treated with 30 mM SRT1720 or 20 µM SRT2183 for 6 h. Protein extracts were analyzed by western blot analysis using antibodies targeting N1IC and β-actin. Activation of SIRT1 by the small molecule SRT2183 reduced the endogenous N1IC protein levels. (B) Lung cancer-derived ECs were cultured in the absence or presence of DLL4 and treated with 20 mM NAM for 6 h. Protein extracts were analyzed by western blot analysis using antibodies against N1IC, SIRT1, and β-actin. Moreover, NAM blockade of SIRT1 activity in lung cancer-derived ECs increased the endogenous N1IC protein levels. (C) HEY1 and HEY2 mRNA levels were measured by real-time RT-PCR using total RNA obtained from lung cancer-derived ECs. The data represent the average of three independent samples obtained at each time point, and each sample was measured in triplicate. Values were expressed as relative arbitrary units following normalization to β-actin mRNA expression. Lung cancer-derived ECs were pretreated with DAPT or solvent (DMSO) and were then replated with DLL4 or solvent for 6 h. * P<0.05 as compared to the control. (D) Lung cancer-derived ECs were cultured in the absence or presence of DLL4 and treated with 20 µM SRT2183 or 30 µM SRT1720 for 6 h. All experiments were performed at least three times (mean±SD). * P<0.05 as compared to the control.
(TIF)

Figure S2 **SIRT1 regulates angiogenic activity in lung cancer-derived ECs.** (A) Lung cancer-derived ECs were transfected with control or SIRT1 siRNAs and cultured with solvent or DLL4 with or without DAPT for the indicated time period. Afterwards, the cell growth was determined using a colorimetric procedure. *indicates significant difference between SIRT1 siRNA ECs and control cells (P<0.05). **indicates a significant difference between the combination treatment (SIRT1 siRNAs, DLL4 and DAPT) and SIRT1 siRNAs plus DLL4 alone (P<0.05). (B) DAPT treatment reversed the impaired sprout elongation in SIRT1 siRNA-treated lung cancer-derived ECs. Representative images and a statistical summary are shown for the results from a three-dimensional in vitro angiogenesis assay with collagen gel-embedded endothelial spheroids transfected with various combinations of control or SIRT1 siRNAs treated with DMSO or 10 M DAPT. Cumulative sprout length was quantified after 24 h.
(TIF)
