# Correction: SIRT1 Regulates Endothelial Notch Signaling in Lung Cancer

**DOI:** 10.1371/annotation/b81b3c0e-108e-479e-b2cf-91aa92623495

**Published:** 2013-04-25

**Authors:** Mian Xie, Ming Liu, Chao-Sheng He

There were errors in the Funding statement. The correct statement is:

This work was supported by grants from the Science and Technology Planning Project of Guangdong Province, China (number 83050), the Medical Scientific Research Foundation of Guangdong Province, China (number A2009265) and Scientific Research Foundation for the Returned Overseas Scholars, Guangzhou Medical University (number 2010 C21). The funders had no role in study design, data collection and analysis, decision to publish, or preparation of the manuscript. 

