# Correction: SIRT1 Regulates Endothelial Notch Signaling in Lung Cancer

**DOI:** 10.1371/annotation/d8a41471-71e5-45a2-bb57-5f565258142f

**Published:** 2013-04-25

**Authors:** Mian Xie, Ming Liu, Chao-Sheng He

There were errors in the author affiliations. The correct affiliations are as follows:

Mian Xie^1,2^, Chao-sheng He^3^, Ming Liu^1,2^

1 China State Key Laboratory of Respiratory Disease, The First Affiliated Hospital of Guangzhou Medical University, Guangzhou, China

2 Guangzhou Institute of Respiratory Disease, The First Affiliated Hospital of Guangzhou Medical University, Guangzhou, China

3 Southern Medical University, Guangzhou, China 

