# Supplementary figures and images for: SIRT1 Regulates Endothelial Notch Signaling in Lung Cancer
Source: PLoS One. 2012 Sep 18;7(9):e45331. doi: 10.1371/journal.pone.0045331 (PMC3445453; doi:10.1371/journal.pone.0045331)

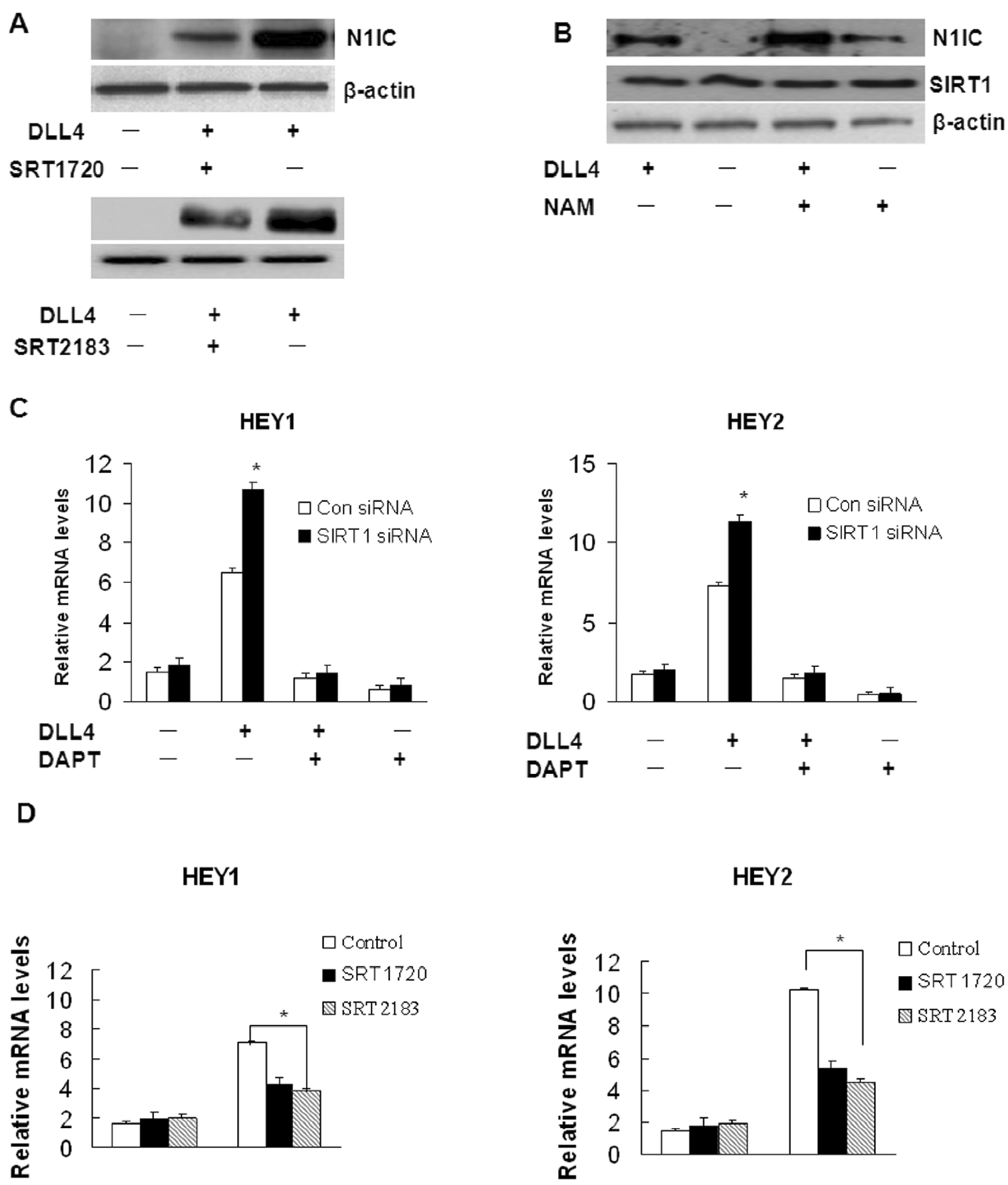

Supplement: Figure S1 — SIRT1 negatively regulates N1IC and controls Notch target gene expression in lung cancer-derived ECs. HEY1 and HEY2 mRNA levels were measured by real-time RT-PCR using total RNA obtained from lung cancer-derived ECs. The data represent the average of three independent samples obtained at each time point, and each sample was measured in triplicate. Values were expressed as relative arbitrary units following normalization to β-actin mRNA expression. (A) Lung cancer-derived ECs were cultured in either the absence or presence of DLL4 and treated with 30 mM SRT1720 or 20 µM SRT2183 for 6 h. Protein extracts were analyzed by western blot analysis using antibodies targeting N1IC and β-actin. Activation of SIRT1 by the small molecule SRT2183 reduced the endogenous N1IC protein levels. (B) Lung cancer-derived ECs were cultured in the absence or presence of DLL4 and treated with 20 mM NAM for 6 h. Protein extracts were analyzed by western blot analysis using antibodies against N1IC, SIRT1, and β-actin. Moreover, NAM blockade of SIRT1 activity in lung cancer-derived ECs increased the endogenous N1IC protein levels. (C) HEY1 and HEY2 mRNA levels were measured by real-time RT-PCR using total RNA obtained from lung cancer-derived ECs. The data represent the average of three independent samples obtained at each time point, and each sample was measured in triplicate. Values were expressed as relative arbitrary units following normalization to β-actin mRNA expression. Lung cancer-derived ECs were pretreated with DAPT or solvent (DMSO) and were then replated with DLL4 or solvent for 6 h. * P<0.05 as compared to the control. (D) Lung cancer-derived ECs were cultured in the absence or presence of DLL4 and treated with 20 µM SRT2183 or 30 µM SRT1720 for 6 h. All experiments were performed at least three times (mean±SD). * P<0.05 as compared to the control. (TIF) [file pone.0045331.s001.tif]

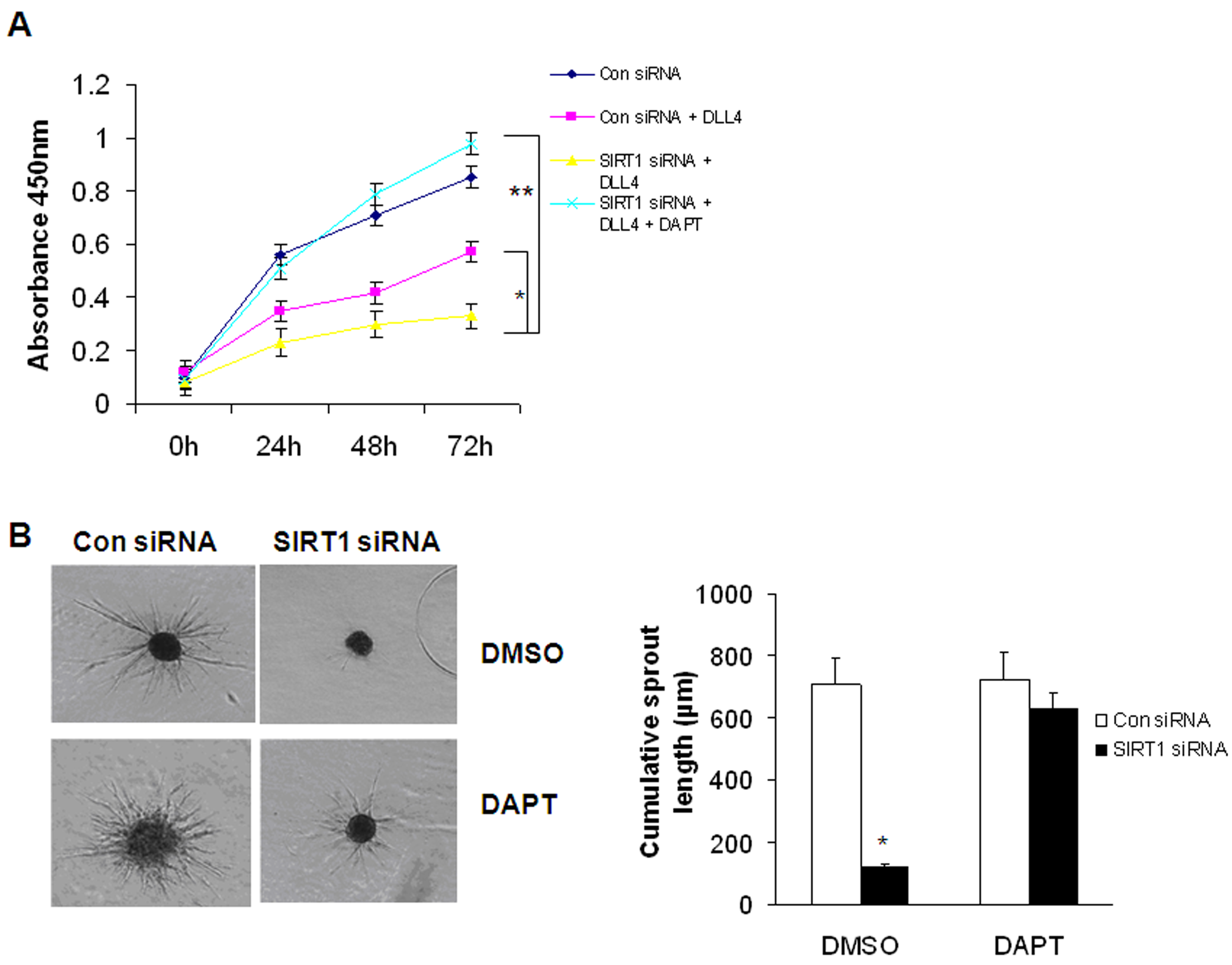

Supplement: Figure S2 — SIRT1 regulates angiogenic activity in lung cancer-derived ECs. (A) Lung cancer-derived ECs were transfected with control or SIRT1 siRNAs and cultured with solvent or DLL4 with or without DAPT for the indicated time period. Afterwards, the cell growth was determined using a colorimetric procedure. *indicates significant difference between SIRT1 siRNA ECs and control cells (P<0.05). **indicates a significant difference between the combination treatment (SIRT1 siRNAs, DLL4 and DAPT) and SIRT1 siRNAs plus DLL4 alone (P<0.05). (B) DAPT treatment reversed the impaired sprout elongation in SIRT1 siRNA-treated lung cancer-derived ECs. Representative images and a statistical summary are shown for the results from a three-dimensional in vitro angiogenesis assay with collagen gel-embedded endothelial spheroids transfected with various combinations of control or SIRT1 siRNAs treated with DMSO or 10 M DAPT. Cumulative sprout length was quantified after 24 h. (TIF) [file pone.0045331.s002.tif]
